# Covid-19 crisis impact on the next generation of physicians: a survey of 800 medical students

**DOI:** 10.1186/s12909-021-02955-7

**Published:** 2021-10-13

**Authors:** Sandrine Passemard, Albert Faye, Caroline Dubertret, Hugo Peyre, Camille Vorms, Victor Boimare, Stéphane Auvin, Martin Flamant, Philippe Ruszniewski, Jean-Damien Ricard

**Affiliations:** 1 Université de Paris, APHP, Hôpital Robert Debré, DMU INOV-RDB, Service de Neurologie Pédiatrique, NEURODIDEROT, UMR 1141 INSERM, Paris, France; 2 Université de Paris, APHP, Hôpital Robert Debré, DMU DM’UP, Service de Pédiatrie Générale, ECEVE, UMR 1123 INSERM, Paris, France; 3 Université de Paris, APHP, Hôpital Louis Mourier, DMU ESPRIT, Service de Psychiatrie, UMR 1266 INSERM, Colombes, France; 4 Université de Paris, APHP, Hôpital Robert Debré, DMU INOV-RDB, Service de Psychiatrie de l’enfant, NEURODIDEROT, UMR 1141 INSERM, Paris, France; 5 Université de Paris, APHP, Paris, France; 6 Institut Universitaire de France (IUF), Paris, France; 7 Université de Paris, APHP, Hôpital Bichat, DMU DREAM, Service d’Explorations fonctionnelles, Physiologie, Centre du Sommeil, CRI, UMR1149, Paris, France; 8 Université de Paris, APHP, Hôpital Beaujon, DMU DIGEST, Service de Pancréatologie et Oncologie Digestive, Hôpital Beaujon, CRI, UMR 1149 INSERM, Clichy, France; 9 Université de Paris, APHP, Hôpital Louis Mourier, DMU ESPRIT, Service de Médecine Intensive Réanimation, IAME, UMR 1137 INSERM, Colombes, France

**Keywords:** Medical students, Volunteering, Covid-19

## Abstract

**Background:**

Many initiatives have emerged worldwide to handle the surge of hospitalizations during the SARS-CoV-2 pandemic. In France, the University of Paris North called on its medical students, whose status makes them integral members of the healthcare staff, to volunteer in their capacity of medical students and/or as nurses/nursing aids in understaffed intensive care units and other Covid-19 services. We attempted to evaluate their commitment, whether the pandemic affected their certainty for the medical profession and career choices, and how they scored their sadness and anxiety levels.

**Methods:**

The University of Paris North took a weekly official census of the involvement of 1205 4th–6th year medical students during the first lockdown in France. Six weeks after the lockdown began (May 4th), an e-questionnaire was sent to 2145 2nd-6th year medical students. The survey lasted 4 weeks and documented volunteering by medical students, the association between the pandemic and certainty for their profession, their choice of medical specialty and factors that influenced sadness and anxiety scores.

**Results:**

82% of 4th–6th year medical students volunteered to continue their internship or be reassigned to COVID-19 units. Of 802 2nd-6th year students who completed the e-questionnaire, 742 (93%) volunteered in Covid-19 units, of which half acted as nurses. This engagement reinforced the commitment of 92% of volunteers to become physicians. However, at the peak of the outbreak, 17% had doubts about their ability to be physicians, while 12% reconsidered their choice of future specialty. Finally, 38% of students reported a score of 7/10 or more on the sadness scale, and 43% a score of 7/10 or more for anxiety. Neither study year nor service influenced sadness or anxiety scores. However, gender influenced both, with women scoring significantly higher than men (*p* < 0.0001).

**Conclusion:**

Medical students of the University of Paris North who made an early and unconditional commitment to help hospital staff handle the pandemic constituted a powerful healthcare reserve force during the crisis. Although the vast majority remained convinced that they want to become physicians, this experience came at a significant psychological cost, especially for women. Alleviating this cost would improve future crisis responses.

**Supplementary Information:**

The online version contains supplementary material available at 10.1186/s12909-021-02955-7.

## Introduction

After China, Europe was the epicenter of the COVID-19 pandemic between January and May 2020, with Italy, France, Spain and the UK being successively devastated by a pandemic wave that subsequently affected America and then the rest of the world. Lockdowns and the strengthening of the healthcare workforce were the two key measures to stop the pandemic for many countries. In France, medical clerkships were suspended on March 17th to comply with government lockdown measures. As in other countries, French medical students were first considered non-essential workers and a potential source of the spread of SARS-CoV-2 [1, 2], and curtailing their movements was logically thought to contribute to the global effort to decrease viral transmission. On the other hand, this has been a critical challenge for the various national healthcare systems, overwhelming healthcare professionals for several months. In order to face the surge of COVID-19 patients in hospitals, an expansion of the workforce was imperative. In New York state, the UK and Italy, final year students were given the choice to graduate early and begin their residency in April instead of July, to participate in the healthcare effort [3, 4]. This redeployment was not conceivable in France since residency is conditional on success in a competitive examination in June.

In France, public health authorities requested the return of reserve or retired healthcare professionals. However, medical and paramedical staff quickly became exhausted, or contracted the virus themselves and had to be replaced, especially in Paris and its administrative region. This was soon a source for concern, and Parisian teaching hospitals, like other French medical source, asked: could medical students join healthcare workers and help out in hospitals? On March 19th, three health institutions in Paris and its region – the Conference of Medical School Deans, the Parisian public hospital administration (Assistance Publique Hopitaux de Paris) and the Regional Health Agency (Agence Régionale de Santé) – decreed that all health students were members of the healthcare professions, whose rights and duties they share, whether or not this care was linked to the pandemic. These students could thus be redeployed to understaffed services or new units set up to deal with the crisis.

**Fig. 1:**
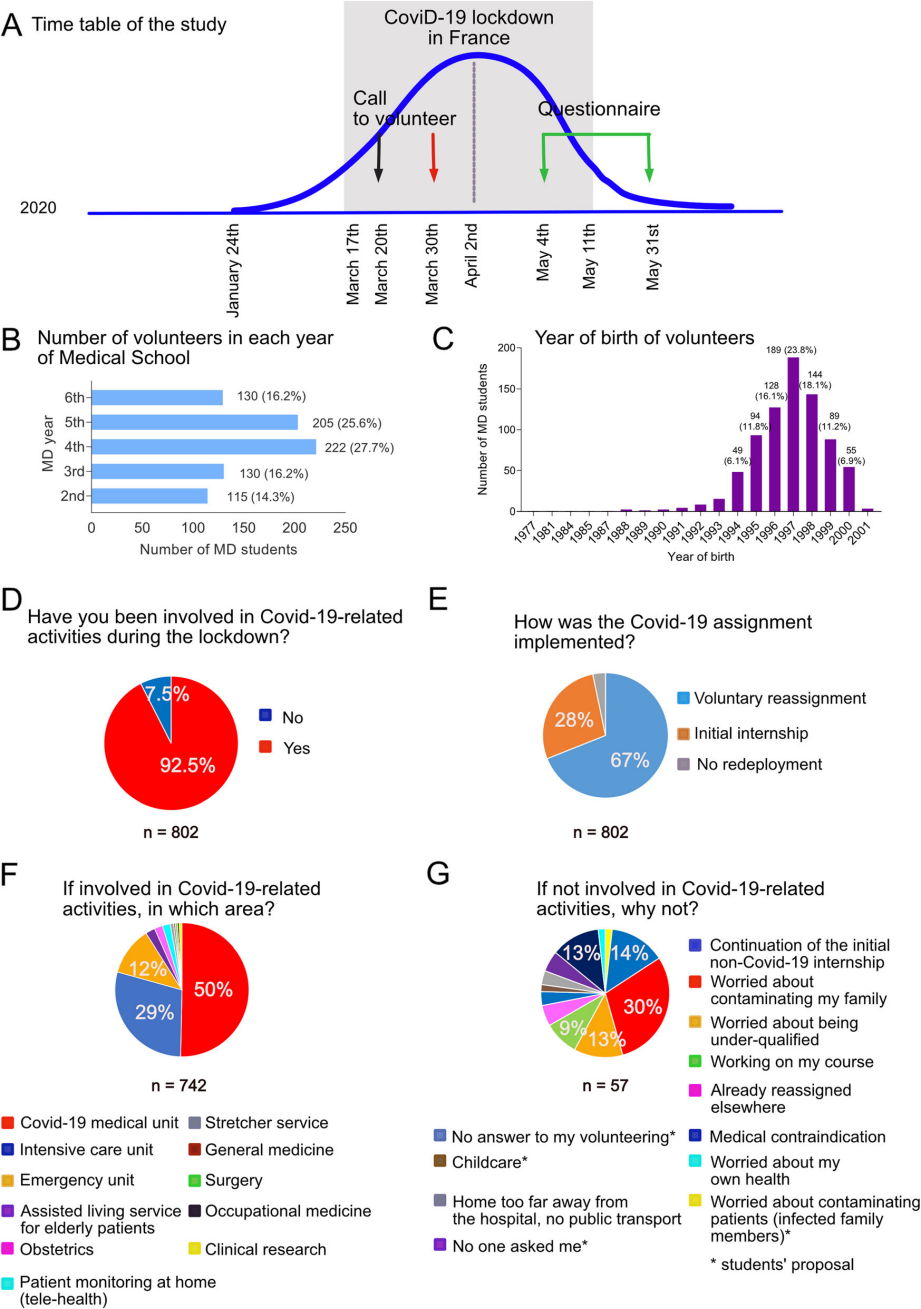
Commitment of medical students with regard to Covid-19 –related activities. **A**: Time table of the study; **B**-**C**: General characteristics of medical students who volunteered in Covid-19 –related activities; **D**-**G**: Distribution of medical students who volunteered in the different Covid-19 –related activities. Source: Medical students survey data

Consequently, since the beginning of the lockdown on March 17th, 2020, the Dean of the medical school of the University of Paris North* decided i) to cancel the first clerkships of the most junior students (2nd and 3rd year) in order to ensure their safety and allow them to lockdown like the rest of the French population, and ii) to let the more experienced 4th- to 6th-year students either to continue their internships in non-COVID-19 services or to volunteer on the COVID-19 frontlines alongside healthcare providers. By March 20th, faced with a health service under pressure and after ascertaining the capacity of both the teaching hospitals and the medical school to prepare and protect them [5], the Dean and teaching staff of the University of Paris North medical school decided to issue a call to all medical students (Fig. 1A) to volunteer as medical students or act as nurses or nursing aids in understaffed intensive care units and other COVID-19 units.

Six weeks after the Dean’s call, how many medical students volunteered and in which departments? What consequences has the outbreak had on the way they consider their training and future career? Did they experience sadness or anxiety? Mindful that i) the role of medical students during the Covid-19 pandemic has been very different across countries and is a matter of debate [1, 5, 6], and ii) this pandemic has been an ordeal even for many hardened personnel [7], it seemed to us essential to examine the impact of the crisis on medical students of the University of Paris who volunteered in Covid-19 units, as well as changes that this crisis induced on their perception of their future profession. Specifically, we aimed to investigate their involvement in Covid-19 units, the impact of this pandemic on their motivation for the medical profession, and their level of sadness and anxiety at the peak of the outbreak.

## Methods, study design and procedures

### Call to volunteer

#### Ethical considerations

Exposing medical students to the risk of Covid-19 infection has been considered and evaluated, both by the French Ministry of Health and its local administrations (Agences Régionales de la Santé), and by the national conference of deans (see Supplementary information, Additional file 1), taking into account current medical and scientific information from around the world.

Four major arguments led to the final decision of the Medical School of the University of Paris North to call on medical students to volunteer:

i) Above all, the priority was to care for all citizens. The need for trained and skilled caregivers was very high, and all caregivers were requisitioned whatever their age.
ii) According to French law, 4th to 6th-year medical students are health professionals. They share the rights and duties of their profession, whether or not care needs are related to the pandemic (see Supplementary information, Additional file 1).
iii) According to Chinese studies, young people were less likely to develop severe forms of Covid-19 than older or retired caregivers [8, 9]. Requisitioning retired or elderly care givers was ethically more questionable than training young volunteers.
iv) The motivation of the students themselves to volunteer was also a determining factor.

#### Process of the call to volunteer

According to the recommendations of both the Conference of Medical School Deans and the Ministry of Health, as described above, the Dean and teaching staff of the University of Paris North medical school decided to issue a call to all medical students (*n* = 2145) encouraging them to volunteer alongside caregivers at teaching hospitals (Fig. 1A). This call was conducted in 2 phases:

- call to volunteer as medical students on the frontlines with patients for 4th–6th year medical students (see Supplementary information, Additional file 2);
- call to volunteer as nurses or nursing aids for 3rd-6th years medical students (see Supplementary information, Additional file 3).

Furthermore, all students, including the most junior ones were encouraged to help i) in teaching hospitals as stretcher bearers, as clinical researchers for COVID-19-related research data collection and entry, or as medical students responsible for the home-monitoring of less severe patients through telemedicine, or ii) in other units close to their home (general medicine, assisted living services for elderly patients …).

### Preparedness

Prior to working as a nurse or nursing aid, medical students who volunteered were trained in the qualifications required for these positions in the teaching hospitals. This training lasted 2 days. During the entire contract period, medical students were supervised by a senior healthcare worker.

Prior to any internship in intensive care units, all medical students are expected to train on the simulation platform of the University of Paris medical school (https://u-paris.fr/ilumens-mieux-former-pour-mieux-soigner/). This rule also applied to all students who volunteered in intensive care units during the outbreak. The aim of this one-day training course was to introduce them to emergency procedures such as cardiac massage, initiation of ventilation and setting up a venous infusion.

Because of the number of different hospitals to which students could be assigned as well as the lockdown and restrictions on travel and the grouping of too many students in the same place, the preparation of medical students was left to the discretion of each medical ward. However, a core set of measures were provided: information regarding the disease and risk of contamination, personal protection equipment, and collective protection measures.

In each Covid-19 unit, students were trained and accompanied daily in the acute management of patients by senior physicians, always working in pairs of “1 student -1 senior physician”. They learned to closely monitor vital signs, recognize clinical symptoms of disease progression (respiratory distress, organ failure, multi-systemic inflammatory syndrome, etc.) and to identify radiological and/or biological indicators of disease aggravation. They were asked to provide regular feedback to their attending physicians and were invited to attend meetings where therapeutic intervention was decided. Medical students did not attend interviews with families.

Medical students who volunteered as students or nurses in COVID-19 units were trained to protect themselves and patients in the same way as caregivers.

### Census of student enrolment

From March 20th, all 4th–6th year medical students (*n* = 1205) were required on a weekly basis to declare their situation to the education office of the University of Paris North: continuation of their internship, voluntary reassignment or lockdown.

### Student survey

The questionnaire was drawn up by a steering committee (the authors) consisting of teaching and healthcare professionals, the chair and members of the Teaching Committee, the Dean’s advisor for Teaching, the Dean himself, the chair of the scientific committee and elected students. The steering committee was responsible for determining research objectives and ensuring that the questions were understandable to the participants and the answers appropriate. The questionnaire was designed to take no longer than 15 min. Before dissemination, the questionnaire was tested by residents to identify misunderstandings or inappropriate answers, and corrections made prior to diffusion.

The questionnaire included 4 demographic questions, 8 questions regarding departments in which students volunteered, the position they held, and for those who did not volunteer, the reasons why. In the second part of the questionnaire a series of 8 questions evaluated changes induced by the outbreak (preference of medical specialty, a fresh look at what would be their future profession). The last 7 questions explored their sadness and anxiety levels using a 10-point Likert-type rating scale (0 = no sadness/anxiety; 10 = unbearable sadness/anxiety), and the tools and/or support network they used to overcome this crisis. A blank questionnaire is available in Supplementary Information, Additional file 4. A selection of medical students’ answer to open-ended questions (Q27) is presented in Supplementary information, Additional file 5.

All members of the steering committee commented on and improved the questionnaire, which was then presented to and approved by the Teaching Committee prior to dissemination to medical students as a Google form by the teaching team. Students were invited to click on the URL link. Students gave their consent to participate by responding to this voluntary and anonymous survey. No return to the participant was possible. The study did not record any personal information capable of identifying a participant, in order to protect anonymity.

The project was approved by the « comité d’évaluation de l’éthique des projets de recherche biomédicale, Paris Nord (N° CER-2020-50).

Forty-one days after the Dean’s call, on May 4th, the University of Paris North medical school sent a link to an e-questionnaire, which included moderated and open-ended questions, to all 2nd-6th year medical students (*n* = 2145). Students received two reminder emails over 4 weeks to request the participation of those who had not yet completed the questionnaire.

All questions and answers to questions are presented in the results section. Access to the questionnaire was possible until May 31st, 20 days after the end of the French lockdown.

### Data analysis

GraphPad Prism version 8.4.3 was used to calculate descriptive statistics, chi-squared, Mann-Whitney U and multiple comparison tests.

The chi-square test was used to compare i) the variable ‘change’ or ‘not change’ in preference of medical specialty after the outbreak, and ii) the variable ‘Yes or No’ in Q20 (at the peak of the outbreak, did you doubt your capacity to be a doctor?) according to sadness and anxiety scores. A Mann-Whitney U test was used to compare sadness and anxiety scores between 2 groups, men and women, and a univariate analysis (nonparametric Kruskal-Wallis test with Dunn’s multiple comparison test) to compare sadness and anxiety scores between 5 groups (2nd to 6th year medical students). The Kruskal-Wallis test was also used to explore the significant association between working in different medical services and sadness and anxiety scores. *P* < 0.05 was considered statistically significant.

## Results

### Which medical students volunteered?

#### Official academic census

The official census of the position of 4th–6th year medical students (*n* = 1205) revealed that only 21% of them decided to remain in lockdown, whereas 79% volunteered to continue their internship or to be reassigned in COVID-19 units (see Table 1). This commitment was much more important for 4th (339/389, i.e. 87%) and 5th year medical students (343/398, i.e. 86%) compared to 6th year medical students (264/418, i.e. 63%).

**Table 1:** Hospital activity of medical students during COVID-19 outbreak

	4th–6th year medical students		4th year medical students		5th year medical students		6th year medical students	
	n	%	n	%	n	%	n	%
*Number of medical students*	1205		389		398		418	
**Hospital activity as medical student**								
In position, on duty	946	79%	339	87%	343	86%	264	63%
Voluntary reassignment in a COVID-19 unit	384	41%	122	36%	167	49%	95	36%
Continuation of initial internship ^a^	562	59%	217	64%	176	51%	169	64%
Reassignment among students released from their initial internship	384	60%	122	71%	167	75%	95	38%
**Hospital activity as nurse or nursing aid**								
Voluntary reassignment in a COVID-19 unit	238	20%	91	23%	119	30%	28	7%
**No hospital activity**								
No voluntary reassignment - Choice for lockdown	259	21%	50	13%	55	14%	154	37%

^a^An important part of the activity of medical departments (Intensive care, Emergency medicine, Infectious diseases, internal medicine ...) has been reclassified as COVID-19 unit during the outbreak, and medical students of these departments have not been offered for changing their assignment

Source: Official census of medical students’ position during the outbreak by the University of Paris North

We did not obtain sufficient information regarding 2nd and 3rd year medical students.

#### Student survey

The link to the e-questionnaire was sent to 2145 medical students 1.5 months after the beginning of the COVID-19 lockdown in France (Fig. 1A); of these, 802 [246 men (30%) and 556 women (70%)], mostly aged between 20 and 26 years, completed the questionnaire, and are referred to hereafter as “responders” (Fig. 1B-C). This distribution was representative of the students enrolled in the medical school at this time [2145 students: 749 men (35%), 1392 women (65%)]. Medical students in the 4th and 5th years accounted for the highest proportion of volunteers (53%). A total of 742 students (92.5%) volunteered to work in units involved with COVID-19 patients (Fig. 1D), mainly through voluntary reassignment (Fig. 1E), as students/trainees (48.5%), nurses/nursing-aids (34.5%) or both (10%). Seven percent of the students maintained their status as medical students and participated either in collecting/recording clinical data for COVID-19 research or in monitoring COVID-19 patients maintained at home using telemedicine. The vast majority of them chose to volunteer in acute care services [COVID-19 medical units (50%), intensive care units (29%), emergency unit (12%), Fig. 1F]. The 7.5% of medical students who did not volunteer (*n* = 60) explained the reason for their choice, except 3: 30% of them were worried about contaminating their family, 2% about contaminating patients, 12% felt underqualified, 14% preferred to continue their initial non-COVID-19 internship, 12% had medical contraindications and 9% preferred to concentrate on their coursework (Fig. 1G).

### Medical students’ commitment as nurses or nursing aids

**Fig. 2:**
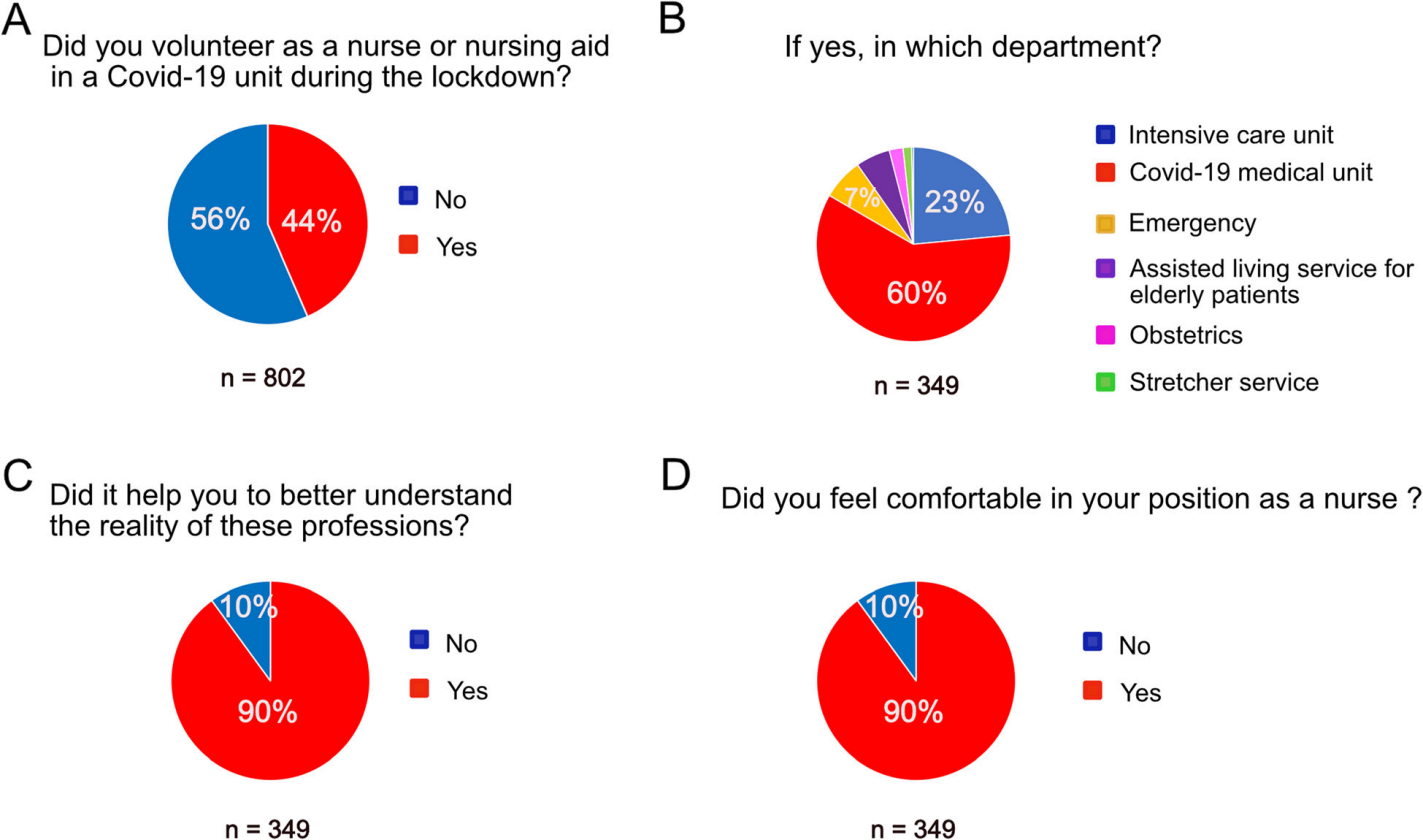
Commitment of medical students as nurses or nursing aids during the Covid-19 outbreak. **A**-**B**: distribution of medical students who volunteered as nurses or nursing aids in Covid-19 units; **C**-**D**: Students' feedback on their experience as nurses or nursing aids. Source: Medical students survey data

Based on the student survey, most of the volunteers were influenced by the shortage of nurses, and responded massively to the Dean’s call to strengthen this professional body. Among the 802 responders, 349 medical students volunteered to work as nurses or nursing-aids (Fig. 2A) did so in COVID-19 medical units (60%), intensive care units (23%), emergency units (7%) or assisted living services for elderly patients (6%, Fig. 2B). Most of them (60%) were 2nd and 3rd year medical students and were in lockdown prior to the Dean’s call; 90% indicated that this experience had helped them to better understand the reality of these professions (Fig. 2C), and 90% also felt comfortable acting as a nurse (Fig. 2D).

### Personal and professional consequences of their commitment

**Fig. 3:**
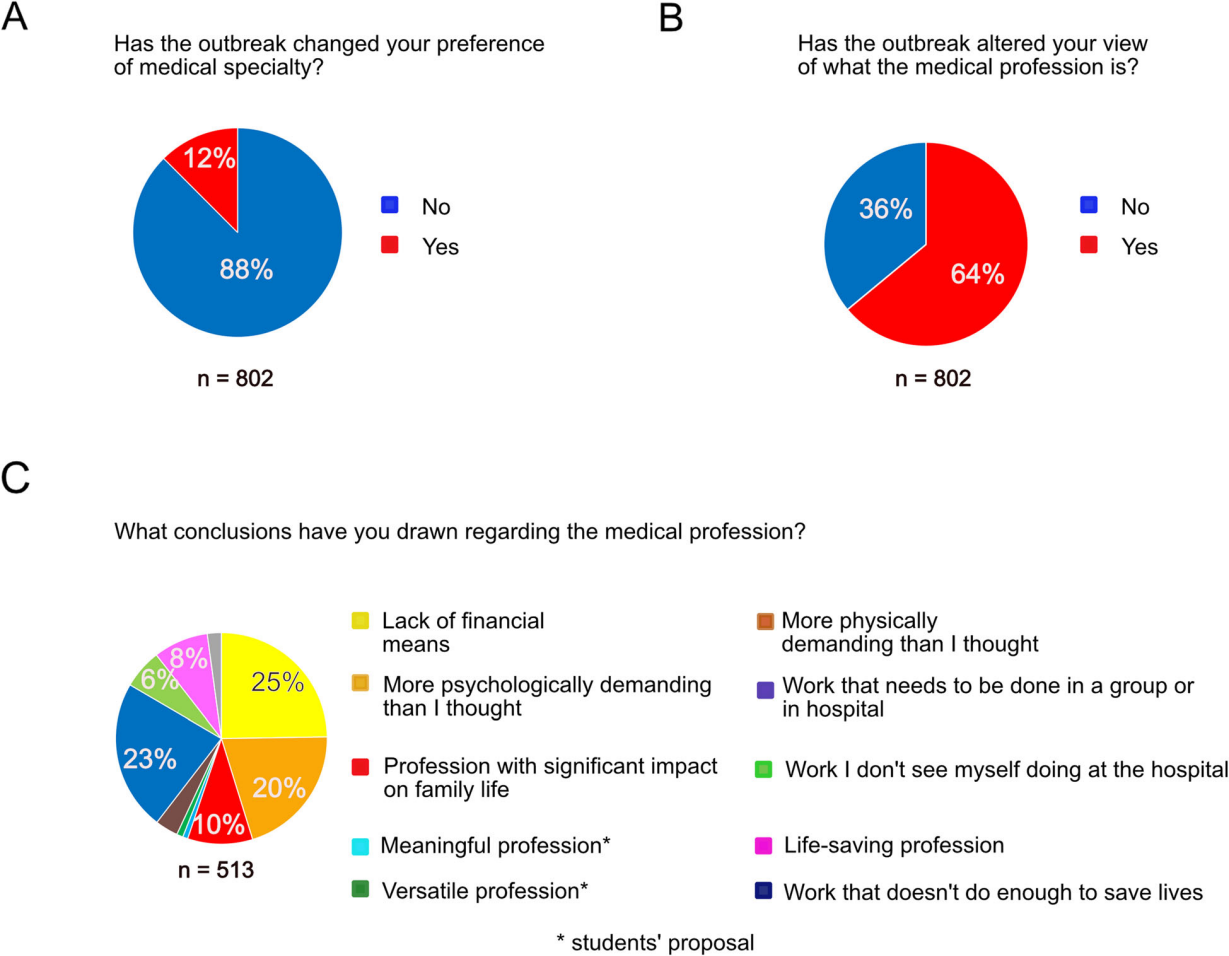
Changes in students’ perception of their future profession during the Covid-19 outbreak. Source: Medical students survey data

The outbreak had multiple consequences for medical students who volunteered. Firstly, it led 12% of them to reconsider their choice of future specialty (Fig. 3A). After the outbreak, a significantly increased percentage of students expressed a preference for intensive care (11.2 to 13.8%, *p* = 0.039, see Table 2). Secondly, the outbreak altered their view of the medical profession (Fig. 3B). A major proportion of those who answered this question (125 out of 513, i.e. 25%) reported that they had become aware of the lack of financial means to protect caregivers and to get the outbreak under control; 20% of them realized that this profession was more psychologically demanding than they had thought and 10% reported that this profession had a significant impact on family life. In addition, 23% thought that they would practice in a group or a hospital. It is worth noting that for 8% of them, this was a life-saving profession (Fig. 3C).

**Table 2:** Impact of Covid-19 on students’ medical specialty preference

	Specialty preference prior to Covid-19 outbreak	Specialty preference after Covid-19 outbreak	*Chi-square test*
Not yet decided	190	188	*ns*
Surgery	145	122	*ns*
Intensive care & Emergency medicine	114	144	*p = 0.039*
General medicine	108	108	*ns*
Pulmonology / Cardiology	40	35	*ns*
Infectious diseases	10	15	*ns*
Other medical specialties	115	110	*ns*
Paediatrics	48	41	*ns*
Psychiatry	20	22	*ns*
Geriatrics	1	5	*ns*
Radiology	10	11	*ns*
Medical biology	1	1	*ns*

Source: Medical students survey data

Students are significantly more likely to prefer intensive care and emergency medicine after their volunteering during the outbreak than before *(p < 0.05, Chi-square test)*

**Fig. 4:**
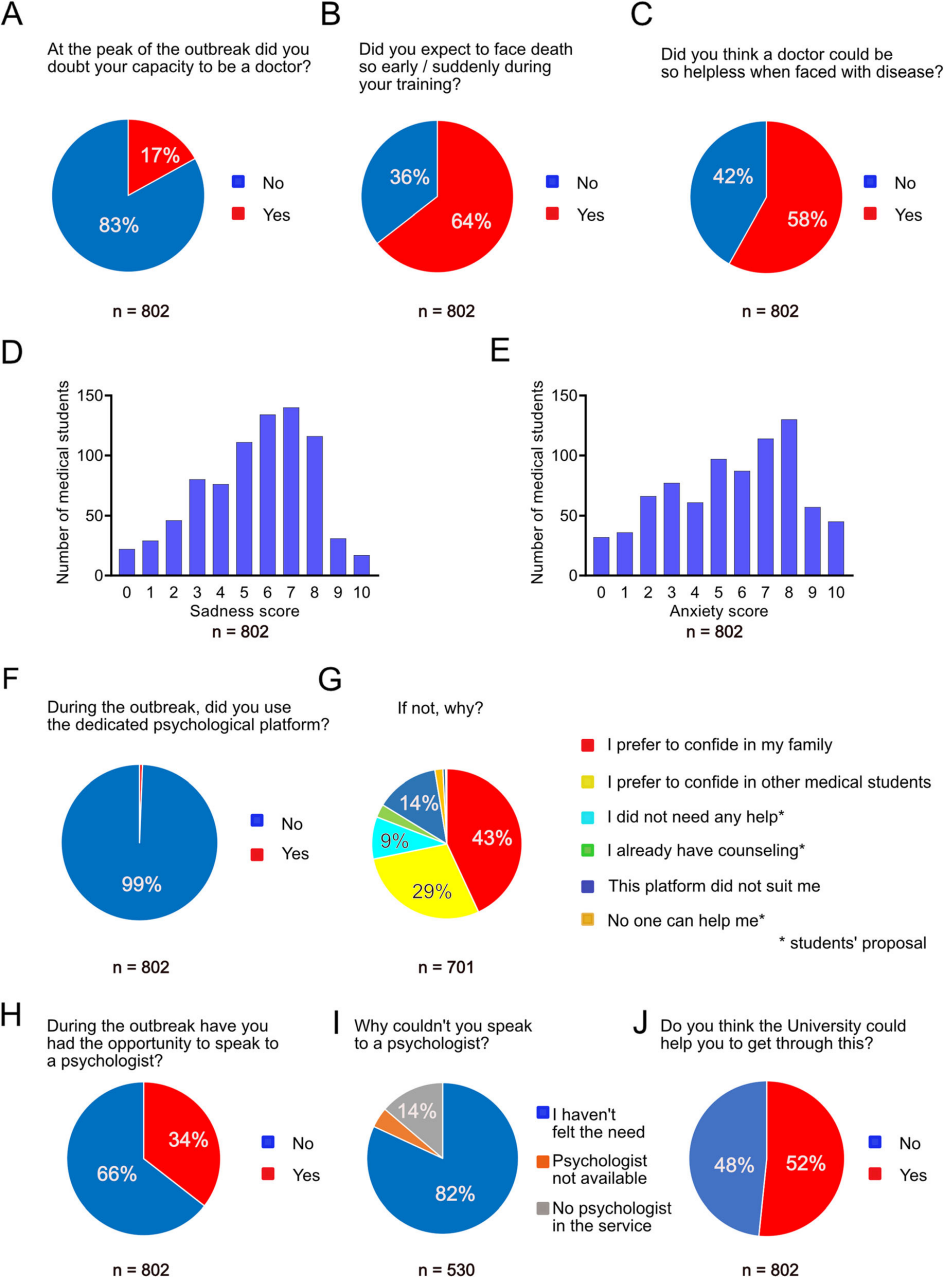
Professional and personal consequences of the Covid-19 outbreak to medical students. **A**-**C**: Students' concerns about the harsh reality of the medical doctor status; **D**-**E**: Sadness and anxiety scores of medical students during the outbreak; **F**-**J**: Personal and institutional resources used by medical students to overcome their psychological distress. Source: Medical students survey data

Next, based on this survey we aimed to know whether the outbreak had shaken their certainty regarding their profession. At the peak of the outbreak, 17% of responders were unsure of their capacity to be physicians (Fig. 4A), explaining that they did not expect to face death so early/suddenly (Fig. 4B) or for doctors to be so helpless when faced with disease (Fig. 4C).

Finally, using numerical Likert-type scales ranging from 1 to 10 to estimate the degree of sadness or anxiety generated by the situation, 38% of responders reported a score of 7 or more on the sadness scale (Fig. 4D), while 43% reported a score of 7 or more for anxiety (Fig. 4E). Sadness and anxiety scores did not vary depending either on the year of medical school (Supplementary Table 1), or the COVID-19 unit (Supplementary Table 2). In contrast, women reported significantly higher scores of both sadness and anxiety than men (Table 3). Finally, medical students who reported significantly higher scores of sadness and anxiety (over 7/10) were those who questioned their capacity to be a doctor (Tables 4 and 5). Although 87.5% of the medical students were aware of the existence of a dedicated psychological platform, few of the responders (5 students out of 802, i.e. 0.6%) used it (Fig. 4F). Of those who did not use it, 701 justified their choice: 14% indicated the fact that the platform did not suit their needs, while 9% reported that they did not feel any need to use it. However, the majority of medical students did not used this platform because they preferred to confide in their family (302/701 i.e. 43%) or in other students (201/701, i.e. 29%, see Fig. 4G). Of the 701 who responded, 20 medical students (2.8%) had already received psychological follow-up or counselling prior to the outbreak. The situation of 12 medical students, i.e. 2%, was preoccupying as they felt that nobody could help them and 3 students out of 701 (0.4%) had dark thoughts. The University of Paris medical school immediately set up a psychological crisis unit for these students in distress. This choice has since been validated by students who believed that the University could help them to get through the crisis (Fig. 4J), by providing more psychological support, improving communication and showing them more recognition. They also suggested constructive courses / activities to be implemented in the future (Supplementary information, Additional file 5). The teaching hospital also suggested during outbreak that medical students, like all caregivers, speak with a psychologist. Out of 802 medical students, 285 (36%) benefited from this service (Fig. 4H). Of those who did not talk to a psychologist, 82% answered that they had not felt the need to do so (Fig. 4I).

**Table 3:** Differences in sadness and anxiety scores between females and males

Factors	Gender	Mean +/− SD	*p*
Sadness score	female	5.887 +/− 2.074	p < 0.0001 ****
	male	4.504 +/− 2.554	
Anxiety score	female	6.016 +/− 2.507	p < 0.0001****
	male	4.496 +/− 2.831	

Source: Medical students survey data

**** denote differences in sadness and anxiety scores between females and males, which are very significant (*p < 0.0001, Mann Whitney test*)

**Table 4:** Impact of sadness score on students’ doubts regarding their capacity to be a doctor

SADNESS SCORE					
Likert score		0 to 6	7 to 10	Total	*Chi squared with 1 degrees of freedom / Two-tailed P value*
At the peak of the outbreak, did you doubt your capacity to be a doctor?	NO	442	224	666	30.446 / *p* < 0.0001
	YES	56	80	136	
	Total	498	304	802	

Source: Medical students survey data

Students with a sadness score of 7 or higher have significantly more doubts as to their being suited to this occupation than those with a score between 0 and 6 (*p < 0.0001, Chi-square without Yates’ correction*)

**Table 5:** Impact of anxiety score on students’ doubts regarding their capacity to be a doctor

ANXIETY SCORE					
Likert score		0 to 6	7 to 10	Total	*Chi squared with 1 degrees of freedom / Two-tailed P value*
At the peak of the outbreak, did you doubt your capacity to be a doctor?	NO	412	254	666	40.092 / *p* < 0.0001
	YES	44	92	136	
	Total	456	346	802	

Source: Medical students survey data

Students with an anxiety score of 7 or higher have significantly more doubts as to their being suited to this occupation than those with a score between 0 and 6 (*p < 0.0001, Chi-square without Yates’ correction*)

## Discussion

To our knowledge, this is the first study combining an official academic census and a voluntary survey that reports on such a large scale and in such detail regarding i) the massive commitment of a large group of 2nd-6th year medical students to work alongside healthcare professionals in contact with patients to face the COVID-19 pandemic, and ii) the experience that students have gained from this commitment.

In addition to the official census, this survey conducted during and immediately after the COVID-19 lockdown in France highlights the important commitment of the medical students of the University of Paris North, regardless of their year in medical school, to work alongside healthcare workers either as medical students, nurses/nursing aids or both. They mainly volunteered in intensive care or emergency units as well as other COVID-19 medical units, 24 h after the Dean’s call.

Commitment was much greater among 4th and 5th year medical students (90.6%) than 6th year medical students (66%). This difference in commitment can be linked to the particular features of medical training in France. Residency training depends on rank in the residency exam, a national competition that occurs at the end of the 6th year of medical school, and for which medical students prepare from the beginning of the 5th year. Commitment was on a voluntary basis, and many 6th year medical students preferred to work on their courses to succeed in the residency exam rather than spending their time volunteering and losing the chance of being well ranked in the competition and being able to choose their specialty.

Many Universities worldwide have advocated the redeployment of students as a vital force to help overstretched health systems, especially for implementation of preventive policies, guidance and services to symptomatic individuals for junior students [3, 10], or as residents for final years students the allowed to graduate early [4, 11]. Although some students expressed a fear of being contaminated or of making serious medical mistakes, being insufficiently prepared if given a choice to return to hospitals [12, 13], various useful and valuable initiatives away from the frontlines have originated from the students themselves in the USA, the UK, Iran, and Canada [14–18]. Nevertheless to our knowledge, few undergraduate medical students have volunteered to be in direct contact with patients in COVID-19 units, except for Danish students who worked as temporary residents, ventilator therapy assistants or nurse assistants [19], American students from Washington state [6] and French students reported here.

The French initiative was made possible by the specific status of French medical students: they are considered healthcare professionals from the first year of medical school, with rights and duties similar to those of healthcare providers. This is really noticeable from their 3rd year clerkship, after their 1 month nurse clerkship in the 2nd year. Working daily at the hospital from the 4th year, French medical students are actually part of the medical staff.

Precisely for this reason, psychological consequences (sadness and anxiety) might be higher in our medical students than in those from other countries who did not directly or physically participate in the pandemic response [20, 21]. For those medical students who stayed at home, as for students in other fields, anxiety was generated by uncertainty, inevitable contradictory instructions, an unsuitable learning environment, delays or advances in examinations and online assessments, consequences of the outbreak to daily life and fear for family and friends. Medical students of the University of Paris North who volunteered faced the pandemic as bravely as healthcare professionals and might have experienced a similar increase in their level of sadness, stress and anxiety. Psychological stress in medical and other healthcare providers has been assessed during and after the pandemic in China, revealing a high prevalence of depression (50.7%), anxiety (44.7%) and insomnia (36.1%) as well as stress-related symptoms (73.4%) in medical staff [22]. In our study, sadness and anxiety levels were assessed using simple Likert-type 0-to-10 rating scales, one of the most common and cited measurement methods to assess behavior in psychology [23]. Such exploratory tools have been reported to have acceptable psychometric properties to measure these two psychological traits for exploratory purposes [24–26], and were used to provide psychological support to students during and after the crisis. This study also highlighted the fact that the only factor influencing sadness and anxiety scores among those tested was gender. Neither the year of medical school (except between 3rd and 6th year medical students) nor more surprisingly, the department in which medical students worked modified sadness/anxiety scores. Given that the medical profession is becoming more and more feminine, Universities must take this parameter into account to support the next generation of physicians. Finally, this study also revealed that medical students with higher sadness and anxiety scores were those who responded that they doubted in their capacity to be a doctor. Sadness and anxiety scores could in no case be interpreted as signs of depression or anxiety disorders, but at the peak of the outbreak they might have reflected a psychological distress in medical students. Taking this into account as well as students’ constructive propositions to overcome this ordeal, the University of Paris has set up a tutorial and discussion groups led by a teaching physician for those who wish to participate.

Being confronted too early and every day for 2 months with patients’ deaths and the powerlessness of physicians also made our medical students realize that this profession lacked financial means, was more psychologically demanding than they thought and had a significant impact on family life. This crisis also led medical students to rethink their medical specialty choice. Significantly more students leaned towards Intensive care or emergency departments. These issues will need to be particularly monitored, and if confirmed in the future, will have to be taken into account by the Ministry of Health, teaching hospitals and medical schools. The career choices of the next generation of physicians will be different from those of current physicians. This pandemic will have left its mark on a whole generation of students, the physicians who will graduate between 2024 and 2030. This may result in changes to public health policies in the future.

This observational study also has limitations: i) the use of a non-validated tool as a Likert scale to assess sadness and anxiety scores, even though it has been widely referenced in the past and is better adapted to our needs than more recent and validated measurement tools [27], ii) the absence of a comparison group for the sadness and anxiety scores, and iii) the fact that this study only reflects the opinion of the survey responders, i.e. 802/2145 or 37.4% of the medical students of the University of Paris North, among whom 742 (92.5% of the responders and 34.6% of all students), volunteered. However, combined with the official academic census which shows that more than 85% of 4rd and 5th years medical students volunteered, this survey seems to us to be a fair reflection of the opinion of the majority of students and reveals strengths:

- They were not afraid to volunteer rapidly to handle a pandemic;
- They experienced team spirit and solidarity with healthcare professionals, helping doctors as well as nurses/nursing aids and were comfortable with their position;
- While this pandemic induced a high level of sadness and anxiety, these students were able to draw upon resources – their family and other medical students for more than 70% of them – to handle it.

## Conclusion

Medical students of the University of Paris North made a commitment very early and without reservations to help hospital staff in their efforts to handle the COVID-19 pandemic, as far as possible while remaining safe. They have been a major part of the COVID-19 response in our country. They have proven that they are capable of working as medical students or as nurses/nursing aids in COVID-19 intensive care and emergency units, other COVID-19 medical units and institutions for elderly patients. In addition, with supervision, they have informed, tracked and monitored less severely infected patients at home using telehealth tools.

This experience as volunteers has reinforced their motivation to become physicians, even if it has come at a significant price in terms of psychological distress. For half of the responders, the medical school has remained a source of support capable of helping them to overcome this ordeal and preparing them to face similar pandemics/crises in the future.

Together, this study demonstrates that medical students, who form an important part of the response to public health crises, might be a fragile population despite their dedication to their work, and provides valuable insights into how they can be better supported in order to improve the efficiency and sustainability of their efforts and reinforcement of their vocation.

*Note: the current University of Paris is the product of a merger between two pre-existing Universities, each with a medical school. Since only one of these was involved in the current study, we have used the term “University of Paris North”, previously Paris Diderot University, to indicate the participating medical school.

## Supplementary Information

**Additional file 1.**

**Additional file 2.**

**Additional file 3.**

**Additional file 4.**

**Additional file 5.**

**Additional file 6.**

## Data Availability

The datasets used and/or analysed during the current study are available from the corresponding author on reasonable request.
