# Supplementary material for: Covid-19 crisis impact on the next generation of physicians: a survey of 800 medical students
Source: BMC Med Educ. 2021 Oct 13;21:529. doi: 10.1186/s12909-021-02955-7 (PMC8511858; doi:10.1186/s12909-021-02955-7)
Supplement: Supplementary file 1 — Additional file 1. [file 12909_2021_2955_MOESM1_ESM.pdf]

Supplementary information – Additional file 1

Covid-19 crisis impact on the next generation of physicians: a survey of 800 medical students

Organisational plan in Ile-de-France for health students[1] during the COVID-19 health crisis March 19, 2020

The health crisis caused by the COVID-19 epidemic is unprecedented, global in scope, will last for many weeks and will have a profound impact on everyone's daily life. Decisions made today, at any level, will have to be adapted to a changing situation. For health students, the following principles and priorities are recalled:

1. The first priority is the care of our fellow citizens. All health students are integrated into health care teams and share their rights and duties, whether or not the care is related to the epidemic.
2. The second priority is containment. All health students who are not immediately needed for care must respect home lockdown and explain to those around them the reasons for locking down the population, as part of their mission to promote public health.
3. The evolution of the crisis in the coming weeks will depend on the population's compliance with barrier measures and containment rules. A situation of overflow of the capacities of the health care system is not completely excluded, especially since a proportion of the health care workers will also be affected by the outbreak. Therefore, it will eventually be necessary to accept a practice of care in degraded conditions and a shift in tasks specific to disaster medicine with the objective of providing our fellow citizens with the best possible care in conditions that have become difficult.

The current reorganization of health services and establishments imposes the partial or total closure of certain activities in favor of absolute or relative emergencies, at the same time as the cessation of teaching and research activities. All this leads to the inactivity of many health students. These students are likely to be redeployed to services under pressure or to new activities set up to deal with the crisis. This requires the organization of redeployment and mobilization of health students at the local and regional levels in order to better coordinate them. The principles of this organization are described below. This organizational plan is applied in accordance with the ministerial instruction on the adjustment of training modalities for health students of March 18, 2020.

[1] The notion of health students refers to future health professionals, as defined in the public health code.

Article 1: Health students (whatever their training and level, including post-graduate residents) are placed at the service of the health care offer in the context of the COVID-19 health crisis.

Article 2: The assignment of a student to a facility, outside of a scheduled internship, is the subject of a commitment act framing the mobilization of the latter.

Article 3: In the care structures[2] which continue their activity without modification, the health students in training remain in function in their service with the appropriate protective equipment, in accordance with the ministerial instructions. For residents, their assignments are extended by one month. However, in particular circumstances, upon a motivated decision by the head of the department or the person in charge of the internship supervision, validated by the crisis unit, the internship may be interrupted.

Article 4: In health care facilities that are partially closed, health care students on placement may be redeployed within the facility by simple decision of the local level (management of the facility on the proposal of the local crisis unit) according to their skills, including after additional training if it is deemed necessary. The opinion of the DES coordinator or the TSP pilot or the training director[3] may be sought.
The institution's management informs the student's redeployment, depending on the case: the training director or the managing university hospital (APHP, Mrs. Hélène Oppetit helene.oppetit@aphp.fr), which is responsible for informing the student's faculty, the DES coordinator or the FST pilot, and the ARS Ile-de-France (ARS-IDF-COVID-etudiantsante@ars.sante.fr). Requests for reassignment of interns between health establishments or outside hospitals are accompanied by an opinion from the coordinator of the DES or the pilot of the FST and are transmitted for information to the CHU and for authorization to the ARS (ARS-IDF-COVID-etudiantsante@ars.sante.fr).

Article 5: All transmissions to the ARS are considered as tacit authorization with immediate effect. The ARS has a period of one week to oppose the reassignments in case of dispute.

Article 6: In the absence of continuation of the activity or redeployment, the health student is in lockdown at home, mobilizable at any time. The management of the establishment informs the student of the lockdown, depending on the case: the managing university hospital, which is responsible for informing the university on which the student depends, the DES coordinator or the FST pilot; the training director, and the ARS. The student can be reassigned to another structure than the initial one, by the managing university hospital and the DES coordinator or FST pilot or training director. The latter will inform the ARS of the reassignment of the students.

Article 7: The DES coordinators or FST pilots or training directors inform the managing university hospital and the ARS of health students who find themselves in lockdown without their care structure or their training supervisor having reported it. Health students are invited to do the same with their managing university hospital and/or training directors.

[2] The notion of care structure concerns the health, medico-social and ambulatory departments.
[3] The notion of training director is generic for all training courses.

Article 8: Health students currently on leave or not assigned to a training course must contact their managing university hospital and their DES coordinator or FST pilot or training director to consider their mobilization. Their personal situation will be evaluated and they may be placed at the service of an institution according to their skills, including after additional training if deemed necessary. The availability interrupted by the participation in the management of the health crisis can be renewed at the end of this crisis, until the end of the period initially planned for this availability. The managing university hospital or the director of training, if necessary, informs the ARS of the mobilization of students on the sites.

Article 9: Residents in their research year must contact their managing university hospital and their DES coordinator or FST pilot to consider their mobilization. Their personal situation will be evaluated and they may be placed at the service of an institution according to their skills, including after additional training if it is deemed necessary. Residents whose research work is oriented towards COVID-19 will be excluded from this measure. The managing university hospital or training director, if applicable, will inform the ARS of student mobilizations on the sites.

Article 10: Residents currently doing inpatient or outpatient internships are authorized, on a voluntary basis, to provide inpatient or outpatient care on other internship sites after agreement from the DES coordinator or FST pilot, who transmits the information to the managing university hospital and to the ARS.

Article 11-: For residents on an inter-university hospital internship or an internship abroad during the next semester, the date on which they take up their duties is also postponed by one month. If lockdown persists after June 1, 2020, the interns in inter CHU or abroad will take their position once the lockdown is over and will remain mobilized in their region of origin in the meantime.

Article 12 - Advanced practice nursing students will continue their internship. If they do not, for whatever reason, they contribute to the supply of care. The training director is informed as well as the ARS.

Article 13: For health students who reach the end of their studies, everything must be done to enable them to access a position as a health professional without further delay. The defense of theses and dissertations at the end of the curriculum or for access to a consolidation phase for residents must be maintained and will take place either behind closed doors or preferably by videoconference. Residents who have not yet defended their thesis and who reach the end of their internship at the end of the current semester are authorized to extend their internship by one month, except in the case of installation or the taking of a hospital position.

Article 14: Pregnant health students and those with chronic pathologies must be in lockdown and, if their health condition allows it, assigned to missions without exposure to patients: remote care activities (e.g. call regulation, telemedicine follow-up, and teleconsultation) or non-care activities (e.g. database management, research, coding, and crisis cell coordination).

Article 15: The managing university hospital and/or the training directorates keep an updated directory of lockdown and redeployed students. This directory is made available to the

Medical schools of Ile de France Universities, medical affairs departments, DES coordinators or FST pilots or training directors, and the ARS.
For health students remaining in lockdown, this directory includes the following minimum information: current and past training, level of training acquired, location of residence, and contact details of the DES coordinator or FST pilot or training director, faculty of origin, e-mail address and cell phone.

Article 16: The Deans, DES coordinators or FST pilots or training directors and the ARS, each for the responsibility that is incumbent upon them, undertake to validate the adapted training models independently of the redeployments and lockdown that may be decided. Specific individual situations will be examined sympathetically. All those involved in redeployment and assignments will ensure that students are valued with a view to their graduation.

Article 17: In this context of crisis, the taking of leave must be limited. However, in order to prevent the risk of burnout, department heads or those responsible for supervising internships will ensure that weekly rest periods are effectively guaranteed and will grant additional leave to those showing signs of professional fatigue. Health students showing signs of burnout should benefit from a work stoppage and the occupational health service will be involved in monitoring these situations. The staff of the health care structures and especially the medical, paramedical and administrative superiors must be particularly vigilant to the risks of burnout of health care students and carry out active prevention policies in the health care structures and for students who remain in lockdown.

Article 18: At the end of the crisis, the health care structures or establishments return to the coordinators of the DES or FST pilots or training directors a summary of the attendance of students (first name and surname, start date, end date, number of hours, any on-call duty, assignments). These summaries are then sent by the coordinator, FST pilot or training director to the organization in charge of monitoring the students. Monitoring of paramedical students is carried out by the training director, whether the student is employed, reassigned to his or her employer, on a scheduled internship, or as a reinforcement of the health care offer. The follow-up of medical, pharmacy, odontology and maieutic students is ensured by their university and that of residents by the managing university hospital.

Article 19: The students of the Health Service of the Armed Forces are waiting for the concertation between the ministries concerned.
The mobilization of health students, their supervisors, DES coordinators or FST pilots or training directors, paramedical and administrative staff has already begun and we would like to thank them warmly for living up to their vocation.

Mr. Aurélien ROUSSEAU, Director General of the ARS Ile-de-France
Mr. Martin HIRSCH, Director General of the AP-HP
Prof. Bruno RIOU, President of the Ile-de-France Conference of Health Deans

Contacts :
CHU manager APHP : Ms. Hélène Oppetit helene.oppetit@aphp.fr ARS Ile-de-France: ARS-IDF-COVID-etudiantsante@ars.sante.fr

Translated with www.DeepL.com/Translator (free version)
