# Supplementary material for: Covid-19 crisis impact on the next generation of physicians: a survey of 800 medical students
Source: BMC Med Educ. 2021 Oct 13;21:529. doi: 10.1186/s12909-021-02955-7 (PMC8511858; doi:10.1186/s12909-021-02955-7)
Supplement: Supplementary file 2 — Additional file 2. [file 12909_2021_2955_MOESM2_ESM.docx]

Supplementary information – Additional file 2

**Covid-19 crisis impact on the next generation of physicians: a survey of 800 medical students**

Paris, March 20th 2020

Dear students,

As the health crisis enters a new phase, we would first like to send you a message of profound thanks. Over the past few days, we have witnessed an unprecedented surge of generosity and solidarity, as so many of you have offered your services to help the teams on the frontline of this crisis. This reminds us that our profession remains above all a gift of oneself, in the service of the weakest. Perhaps we sometimes forget this, but it is what drives us deep down. We can assure you that this is very comforting.

Wherever you are, whatever you do, you will contribute to the resolution of this crisis. For some of you, especially the youngest, this contribution will consist of respecting the lockdown and sharing health-related messages to your relatives, which are essential to break the chain of transmission. For others, it will consist of helping the emergency teams in intensive care units, in the emergency room or in dedicated sectors. Others will participate in research, and still others in the continuity of care for patients with other pathologies whose treatment cannot be postponed. There is no big or small contribution, but a battle to be fought together. Some have offered to help in emergency structures and have not yet been mobilised. They may feel a certain frustration, in line with their desire to help. The battle will be long, the teams will have to be reinforced and renewed. We cannot be too many to resolve this crisis, and you will all be mobilised at some point.

We are also aware that, over time, some of you, and some of us, will need support. What we are about to experience will not leave you emotionally or psychologically indifferent, and we are interested in accompanying you if you feel the need. Professor Revah-Lévy's DIADE team will coordinate a response unit, which will accept requests made by those who wish to do so via a dedicated telephone number, with a secondary return call from a member of his team for a telephone interview. It is also possible that this crisis is causing you financial problems, which you can report to us and which we will pass on to the University of Paris, which has been asked to provide assistance to students in difficulty.

As you know, the management of the epidemic requires a major reorganisation of our offer of care in hospital structures. This includes increasing emergency room capacity, increasing the number of intensive care beds in existing departments and by extension in related structures (recovery rooms, operating theatres), and increasing the capacity to receive patients who do not require resuscitation in infectious disease departments, pulmonology departments or in newly created units (Covid+ units). The creation of these Covid+ structures is possible thanks to the reorganisation of medico-surgical activity dictated by the situation: the postponement of cold surgery procedures, the postponement of programmed hospitalisations in the medical sector, and the implementation or reinforcement of remote medical care (teleconsultations, home care).

This has a direct impact on your practical training, with some student facilities closing or significantly reducing their activity, and others increasing theirs. As the transition to stage 3 took place while you were in the middle of a placement, we had to urgently review our organisation. Thus, depending on their placement sites, some students were released from their obligations, while others were kept on. Released students have the possibility of being reassigned to other structures, on a voluntary basis only. We are centralising requests for reinforcements and call on you regularly using dematerialised tools. Some departments also call on you directly, often because you have been previously assigned to them, and we approve of this reassignment after verifying that you have been released from your previous placement. In spite of this, we do not necessarily have a completely exhaustive view of movements, and next week we will ask you to tell us, via a dematerialised questionnaire, your current place of assignment. Thank you for making the effort to fill it in. It is very important for us to be able to declare your current activity.

Students who are not reassigned must respect the lockdown principle imposed on the general population, while waiting to be mobilised if necessary. For DFASM1 and 2 students who have been released from their current placement, reassignment is on a voluntary basis only. On the other hand, our supervisory authorities tell us that DFASM3 students are likely to be requisitioned in the coming weeks (see attached document). For reasons of efficiency, we have decided to keep the students at their posts until further notice. There will therefore be no rotation at the beginning of April. This may cause individual problems, which we will consider on a case-by-case basis. Finally, the distribution of students among different structures does not allow for the organisation of validation exams at the end of the placement, and we will ask the heads of department to effect this validation on the basis of the assessment made on a daily basis by those who have supervised you.

We would also like to inform you of the possibility of extending your assistance through non-medical missions indirectly involved in care, such as stretchering. This is independent of your training as an extern and is done in the form of an employment contract established by the hospital establishment.

Concerning the theoretical training, in the absence of teaching dispenses on faculty sites, we have asked all the teachers to make available to the students, via the Moodle platform, a commented version of the course material, in the form of a written note next to each slide. We will be vigilant in the weeks to come to ensure that this pedagogical effort is really implemented. All additional initiatives allowing teacher-student interaction are encouraged (discussion forums, for example).

We can already announce that it will not be possible to maintain exams on the dates initially planned, either for the faculty exams or for the ECN. The resumption of university activities in general, and of exams in particular, is suspended until the faculty reopens. This will be decided by the supervisory authorities, depending on the evolution of the epidemic. As an indication, we could envisage holding the DFASM exams at the end of June. A new date for the ECN is under consideration, probably in July.

To each and every one of you, we reiterate our thanks and wish you all the courage you need in these difficult times, which underline the magnificent career choice we have all made. The now famous "8 p.m. concerts", which are also intended for you, bear witness to the gratitude of the population towards all those who devote themselves to it. Let us continue in this way, and do not hesitate to come back to us, directly or through your elected representatives, with any questions or requests.

With kind regards to all of you.

Prof. Philippe RUSZNIEWSKI, Dean of the UFR Paris Nord

Prof. Martin FLAMANT, Assessor for Teaching

Prof. Abdellatif TAZI, Vice-Dean

Prof. Albert FAYE, President of the Teaching Commission

Prof. Damien ROUX, vice-president of the Teaching Commission

Prof. Caroline DUBERTRET, President of the Placement Commission
