# Supplementary material for: Covid-19 crisis impact on the next generation of physicians: a survey of 800 medical students
Source: BMC Med Educ. 2021 Oct 13;21:529. doi: 10.1186/s12909-021-02955-7 (PMC8511858; doi:10.1186/s12909-021-02955-7)
Supplement: Supplementary file 3 — Additional file 3. [file 12909_2021_2955_MOESM3_ESM.pdf]

Supplementary information – Additional file 3

Covid-19 crisis impact on the next generation of physicians: a survey of 800 medical students

UFR MEDECINE PARIS NORD

Université de Paris

Paris, 30th March 2020

Dear students,

With the changing health situation, we need to further strengthen and optimise the health care offer. It is becoming increasingly clear that the main limiting factor is human, and concerns paramedical staff.

This is what led us yesterday to call for the mobilisation of all of you to be hired as acting nurses for one month.

The contracts are part-time hospital jobs, corresponding to 21 hours per week.

For students who are currently released from any externship, this working time is not longer than that of the placement which they no longer have.

We are aware, however, that students currently in post or reassigned may find undertaking additional paramedical duties too great a burden to bear.

Under these circumstances, we are offering those who wish to do so the opportunity to convert their current externship into a part-time paramedic position in the hospital where they are currently posted.

Once you have signed the contract, you will either be kept in the department where you are or redeployed. The only particular request is that students continue to fulfil the on-call schedule of their department if they have one.

All this cannot be done without some overall visibility of intentions. Therefore, we would be grateful to you all for responding to this very short Google Form.

https://docs.google.com/forms/d/e/1FAIpQLSfdhZ4ky8EzZJirqydLtRLchaeb5undgZXJfMviWaC_GBXIeQ/viewform?usp=pp_url

We need to move very quickly, as the situation is becoming critical. Any effort, however small, is crucial.

Thank you for your mobilisation.

Pr RUSZNIEWSKI Philippe, Dean of the UFR Paris Nord

Pr FLAMANT Martin, Assessor for Teaching
