# Supplementary material for: Covid-19 crisis impact on the next generation of physicians: a survey of 800 medical students
Source: BMC Med Educ. 2021 Oct 13;21:529. doi: 10.1186/s12909-021-02955-7 (PMC8511858; doi:10.1186/s12909-021-02955-7)
Supplement: Supplementary file 4 — Additional file 4. [file 12909_2021_2955_MOESM4_ESM.docx]

Supplementary information – Additional file 4

**Covid-19 crisis impact on the next generation of physicians: a survey of 800 medical students**

**BLANK QUESTIONNAIRE**

This questionnaire is aimed at medical students who have volunteered to work with Covid-19 patients or to provide assistance to departments that need it. We are grateful for your commitment. We would like to conduct a survey to find out what impact this particular experience may have had on your training course and also to gather your needs. This questionnaire is of course offered anonymously.

**Tell us about yourself**

Q1) What year of medical school are you in? (one answer only)

2^nd^ year □ 3^rd^ year □ 4^th^ year □ 5^th^ year □ 6^th^ year □

Q2) Are you?

A man □ A woman □

Q3) What is your year of birth?

______________

Q4) Do you have any caregivers (medical or non-medical staff) in your family?

Yes □ No □

**As a medical student**

Q5) Have you been involved in Covid-19 related activity during the lockdown?

Yes* □ No □

*If yes, in which area? (one answer only)

Intensive care unit □ Covid-19 medical unit □ Emergency unit □ Surgery □

Obstetrics □ Assisted living service for elderly patients □ Stretcher services □

General medicine □ Occupational medicine □ Patients monitoring at home □

Clinical research □

Q6) How was the Covid-19 assignment implemented? (one answer only)

Voluntary reassignment □ Initial internship □ No redeployment □

Q7) Were you involved in providing information to families about their hospitalised relatives?

Yes □ No □

Q8) To which missions were you assigned? (several possible answers)

□ Patient care:

□ As a 4^th^ to 6^th^-year student

□ As a caregiver (nurse, nursing aid)

□ Participation in Covidom (Home monitoring for Covid-19 patients) □

□ Participation in providing information to families and in reporting results of medical exams

□ Other

**You undertook paramedical duties during the Covid epidemic**

Q9) Did you volunteer to work as a nurse or nursing aid in a Covid-19 unit during the lockdown?

Yes* □ No □

*If yes, in which department? (one answer only)

Intensive care unit □ Covid-19 medical unit □ Emergency unit □ Surgery □

Obstetrics □ Assisted living service for elderly patients □ Stretcher services □

General medicine □ Occupational medicine □

Q10) Has working as a nurse or nursing aid help you better understand the reality of these professions?

Yes □ No □

Q11) Did you feel comfortable in your position as a nurse?

Yes □ No* □

*If not, why? *[Open-ended question]*

**You did not commit in a Covid19 department**

Q12) If you were not involved in Covid19 related activities, is it because (several possible answers):

- You have continued your initial non-Covid-19 internship?

Yes □ No □

- You wanted to work on your course?

Yes □ No □

- You worried about your own health?

Yes □ No □

- You worried about being under-qualified?

Yes □ No □

- You worried about contaminating your family?

Yes □ No □

- Because no one asked you?

Yes □ No □

- Because you had medical contraindication?

Yes □ No □

- Because you was already reassigned elsewhere?

Yes □ No □

- Because you were lockdown far away from the hospital, without any access to public transport?

Yes □ No □

- Other? □ Precise :

**Consequences of the outbreak to your future career**

Q13) What specialty were you planning to pursue prior to the outbreak? (one answer only)

Surgery □ Pulmonology / Cardiology □ Geriatrics □ Intensive care □

Paediatrics □ Infectious diseases □ Radiology □ Other medical specialties □

General medicine □ Psychiatry □ Emergency medicine □ Medical biology □

Not yet decided □

Q14) Has the outbreak changed your preference of medical specialty?

Yes □ No □

Q15) After the outbreak, what specialty do you plan to pursue? (one answer only)

Surgery □ Pulmonology / Cardiology □ Geriatrics □ Intensive care □

Paediatrics □ Infectious diseases □ Radiology □ Other medical specialties □

General medicine □ Psychiatry □ Emergency medicine □ Medical biology □

Not yet decided □

**Impact of the outbreak on your future profession**

Q16) As the outbreak altered your view of what the medical profession is?

Yes* □ No □

*if Yes, what conclusions have you drown regarding the medical profession? (several possible answers)

□ Profession more physically demanding than I thought

□ Profession more psychologically demanding than I thought

□ Lack of financial means

□ Work I don’t see myself doing at the hospital

□ Profession that needs to be done in a group

□ Profession that needs to be done in hospital

□ Life-saving profession

□ Not enough life-saving profession

□ Profession with significant impact on family life

□ Other

Q17) Has the outbreak strengthened your motivation for the medical profession?

Yes □ No □

Q18) Did you expect a doctor could be so helpless when faced with disease?

Yes □ No* □

*if not, why? *[Open-ended question]*

Q19) Did you expect to face death so early / suddenly during your training?

Yes □ No □

Q20) At the pick of the outbreak, did you doubt your capacity to be a doctor?

Yes □ No □

**What did you feel during the outbreak?**

Q21) During the outbreak, did you feel: (several possible answers)

- Powerless?

Yes □ No □

- Like you belonged?

Yes □ No □

Q22) During this pandemic, can you estimate the intensity of your feelings of sadness on a scale of 0 to 10? (0 = no feeling of sadness; 10 = unbearable feeling of sadness) (one answer only)

1. □ 1 □ 2□ 3□ 4□ 5□ 6□ 7□ 8□ 9□ 10□

Q23) During this pandemic, can you estimate the intensity of your feelings of anxiety on a scale of 0 to 10? (0 = no anxiety; 10 = unbearable anxiety) (one answer only)

1. □ 1 □ 2□ 3□ 4□ 5□ 6□ 7□ 8□ 9□ 10□

Q24) Are you aware of the existence of the DIADE platform (a psychological support platform)?

Yes* □ No □

*if Yes, did you use this dedicated platform?

Yes □ No □

Q25) Did this platform help you?

Yes □ No* □

*If not, why? (one answer only)

□ This platform did not suit me

□ I don’t wish to share my experiences with a strenger

□ I prefer to confide in my family

□ I prefer to confide in other medical students

□ Other:

Q26) During the outbreak, have you have the opportunity to speak to a psychologist?

Yes □ No* □

*if Not, why?

□ No psychologist in the service

□ Psychologist not available

□ I haven’t felt the need

Q27) Do you think the University could help you to get through this?

Yes* □ No □

*if Yes, How?  *[Open-ended question]*
