# Supplementary material for: Covid-19 crisis impact on the next generation of physicians: a survey of 800 medical students
Source: BMC Med Educ. 2021 Oct 13;21:529. doi: 10.1186/s12909-021-02955-7 (PMC8511858; doi:10.1186/s12909-021-02955-7)
Supplement: Supplementary file 5 — Additional file 5. [file 12909_2021_2955_MOESM5_ESM.docx]

Supplementary information – Additional file 5

**Covid-19 crisis impact on the next generation of physicians: a survey of 800 medical students**

**Q27: How do you think the University could help you to get through this ordeal?**

**Selection of medical students’ responses to Question 27 “How do you think the University could help you to get through this ordeal?”** (Translated from French by [www.DeepL.com/Translator](http://www.DeepL.com/Translator), then corrected)

***Request for a reduction in university exams***

To be accommodating in terms of exam grades, as the situation has led us to work less on our classes. *4^th^ year medical student*

Minimizing the pressure of exams and really evaluating us on our knowledge and not on MCQs with tricky details. *2^nd^ year medical student*

Postponing the date of the exams. *4^th^ year medical student*

Cancelling the second semester exams. *2^nd^ year medical student*

Taking advantage of the situation to continue to put teaching aids on Moodle platform in the form of commented slides and videos. This is great, but it is sad that we had to wait for Covid-19 to have this in 2020... It is much better adapted to our already very restrictive schedules as opposed to face-to-face courses... It would be really nice if this could continue and be generalized to all subjects. *5^th^ year medical student*

***Request for more psychological support***

By setting up psychological support in a location away from the university, so that students are not anxious about their peers seeing them. *3^rd^ year medical student*

Possibly assigning a psychiatrist from one of the university's hospitals to listen to students who feel the need. *4^th^ year medical student*

As is already the case to some extent, putting into place tools for listening to students in the form of discussion groups or psychologists, because it is easy to feel 'alone' at different moments and not always easy during training. *4^th^ year medical student*

A psychological follow-up should be systematically set up for medical students, pandemic or not. *5^th^ year medical student*

Being more concerned about our mental health from the beginning of our studies. *5^th^ year medical student*

Systematic interviews with psychologists once during the clerkship to screen to diagnose anxiety-depression syndromes and to allow students to speak freely. Daring to ask for help by oneself is very difficult. *4^th^ year medical student*

***Request for more communication / compassion / reassurance or support***

The university can play its role in ensuring that students are well trained and that we feel more at home in the hospital. *4^th^ year medical student*

Good communication, caring and support. *4^th^ year medical student*

I think that the university could have been more present at the beginning of the pandemic when we really felt left out by the university, which had given us little information. We were really lost, which increased my anxiety level. *5^th^ year medical student*

Ensuring proper vacation time during our 2nd semester internships (as well as the assurance of being allowed to take vacation time AFTER the exams). *4^th^ year medical student*

Being forgiving and understanding with regard to the difficulties of the period, the stress experienced by students both in hospital and alone at home facing uncertainties about our exams and the lockdown. *3^rd^ year medical student*

The messages of encouragement were welcome and I think there were a lot. The fact that the faculty encouraged student mobilization without making it mandatory created, in my opinion, a greater willingness to help because it appealed to our personal values (and their conflicts) and what we want to become in this profession.

In addition, the provision for online book consultations, the possible loans, and the DIADE platform were appreciated. *6^th^ year medical student*

The university must be a support for students in this period, offering them a framework that allows them to reconcile their intention to help in the hospital services and to manage their academic deadlines and the stress associated with them (exams and competitions). This requires not only flexibility and responsiveness, but also confidence in the students and their intentions. *5^th^ year medical student*

Understanding, tolerance, patience. *6^th^ year medical student*

Through messages of support and accompaniment. *6^th^ year medical student*

***Request for more recognition***

The university has already done this, by making the listening platform available and showing its appreciation by email, by being flexible on the dates of exams, by allowing evaluations to be kept as the students wanted (with progressive cases and not only MCQs). This has to continue, it is good! It is a pity that it has taken a pandemic to realize that the hospital relies on students, and that we deserve this recognition even in non-Covid times. *4^th^ year medical student*

Revalorise clerkship positions. *3^rd^ year medical student*

To valorise our commitment in curriculum. *4^th^ year medical student*

By emphasizing that we are not just ordinary students, but future caregivers and that we have a full role to play in exceptional situations such as this. I felt that the university fulfilled its role and I felt supported by the faculty. I wanted to thank everyone who made it so easy for us to be work in Covid units. I know that this was not necessarily the case in other universities where students had to handle the process alone. *5^th^ year medical student*

Recognition! Living up to our commitment. More consideration! More empathy! Less ideology and more connection to the reality of our daily lives. *5^th^ year medical student*

***Constructive proposals from medical students***

Training to approach situations of medical impasse or death; experience-sharing seminars in specialties highly exposed to death; proposal for faculty psychologists who would be more flexible in terms of availability and financially more affordable than those available privately... *3^rd^ year medical student*

We enter medical school when we are only 17 years old while we are still building ourselves, and then we learn that it is normal to restrain recalcitrant patients, it is normal to attend a death, it is normal to see destitute people.

However, the only people we can talk to about all this are our friends who are also just students.

Even if we can talk about it to the doctors in our departments, they are still our "superiors".

It would be interesting to be able to talk about it with doctors for 15 students and just talk person to person. To realize that our elders also feel helpless and are living through the same as us. And especially to realize that everything we see is not normal. *5^th^ year medical student*

I was very lucky in my department, because we were very well supervised by our bosses and by a psychological consultation to help us inform the families. This kind of training (how to break the news, body language, how to react to this or that situation, how to protect yourself psychologically as a caregiver from difficult situations) is extremely beneficial. Could they be offered as electives in the lecture hall or in a practical class? The "human" training is indeed very absent in medical studies, which are mainly centred on knowledge and not on how to be and how to accompany the families and patients. *4^th^ year medical student*

As the university already does, by sharing contacts to be able to talk and express ourselves. Create forums etc. *3^rd^ year medical student*

Anonymous testimonials made available to students and possibility to testify. *4^th^ year medical student*

A MOOC on health crisis management, defining a clear role for clerks in times of crisis, earlier training on communication with families, how to deal with deaths, sharing the experiences of our seniors. *4^th^ year medical student*

A post-pandemic lecture hall meeting or a video of university leadership to take stock and really realize what we just went through. *4^th^ year medical student*

I would find relevant the idea that each student have a "tutor" during the whole duration of his/her studies (Fellow, Professor...), as for nursing students. This is to enable exchanges on practices, benefit from advice etc. *3^rd^ year medical student*

Recap of the last 2 months to have an overview of Covid, treatments, care, how to manage a health crisis situation, what we have accomplished as caregivers. Limitations? Areas for improvement. *4^th^ year medical student*

By including training on emotional management, distancing, confronting death and illness. *2^nd^ year medical student*

With courses more focused on the doctor-patient relationship and how physicians sometimes have to deal with feelings of helplessness. Organize more conferences with doctors so that they can share their experience and problems so that from the 2nd year onwards, students can get away from the idealistic vision of the medical profession, that of the saviour, the one who can easily face everything. I also had the opportunity to attend a meeting between the caregivers and the mobile palliative care team to discuss the management of Covid19 patients in geriatrics and it was very interesting, it made me understand and analyse the end of life from another angle --> it would be beneficial for everyone to take advantage of it from the beginning of their studies. *2^nd^ year medical student*

Organize a lecture hall meeting/feedback communication: to understand what was put in place, why it took so long, why the situations experienced and the media coverage were so divergent. How did the APHP organize itself? Where was the money found for the creation of additional beds...? *6^th^ year medical student*

Yes, as you are already doing with the DIADE platform and perhaps by organizing meetings with students and doctors to talk about their experience for those who need it.

By having courses on crisis management for students in clerkships to understand how they can really be useful in terms of their abilities.

Also having training on announcements to patients and relatives as it was quite difficult to learn this in the field +++. 5^th^ year medical student
