# Supplementary material for: Covid-19 crisis impact on the next generation of physicians: a survey of 800 medical students
Source: BMC Med Educ. 2021 Oct 13;21:529. doi: 10.1186/s12909-021-02955-7 (PMC8511858; doi:10.1186/s12909-021-02955-7)
Supplement: Supplementary file 6 — Additional file 6. [file 12909_2021_2955_MOESM6_ESM.docx]

Supplementary Table 1

Differences in sadness and anxiety scores according to the year of medical school

| Factors | Years of medical school | Mean +/- SD | *p* |
| --- | --- | --- | --- |
| Sadness score | 2nd | 5.517 +/- 2.335 | *p=0.4768, ns* |
|  | 3rd | 5.130 +/- 2.549 |  |
|  | 4th | 5.590 +/- 2.241 |  |
|  | 5th | 5.574 +/- 2.202 |  |
|  | 6th | 5.357 +/- 2.357 |  |
| Anxiety score | 2nd | 5.181 +/- 2.705 | *p=0.0033*** |
|  | 3rd | 4.962 +/- 2.690 |  |
|  | 4th | 5.698 +/- 2.813 |  |
|  | 5th | 5.618 +/- 2.521 |  |
|  | 6th | 6.116 +/- 2.677 |  |

Source: Medical students survey data.

**** denote differences in sadness and anxiety scores according to the year of medical school of the students, which are significant *(p<0.05,* *Kruskal-Wallis test, followed by Dunn’s multiple comparisons test when significant)*

Supplementary Table 2

Differences in sadness and anxiety scores according to the Covid-19 service

| Factors | Services | Mean +/- SD | *p* |
| --- | --- | --- | --- |
| Sadness score | Medicine | 5.517 +/- 2.335 | *p=0.3396, ns* |
|  | Intensive care | 5.130 +/- 2.549 |  |
|  | Emergency | 5.590 +/- 2.241 |  |
|  | Teleconsulting | 5.574 +/- 2.202 |  |
|  | No assignment | 5.357 +/- 2.357 |  |
| Anxiety score | Medicine | 5.476 +/- 2.711 | *p=0.8028, ns* |
|  | Intensive care | 5.484 +/- 2.691 |  |
|  | Emergency | 5.616 +/- 2.701 |  |
|  | Teleconsulting | 6.235 +/- 3.052 |  |
|  | No assignment | 5.672 +/- 2.781 |  |

Source: Medical students survey data.

**** denote differences in sadness and anxiety scores according to the service in which the students worked, which are significant *(p<0.05,* *Kruskal-Wallis test, followed by Dunn’s multiple comparisons test when significant)*
